# LEVERAGING DRUG-DRUG INTERACTIONS FOR PHARMACOLOGICAL EXTENSION OF HEALTHY LIFESPAN

**DOI:** 10.1093/geroni/igz038.2296

**Published:** 2019-11-08

**Authors:** Jan Gruber

**Affiliations:** Yale-NUS, Singapore, Singapore

## Abstract

Traditional approaches aimed at delaying or preventing age-dependent diseases view each disease as a distinct entity, resulting from separate pathophysiological chains of events. However, it is becoming increasing clear that even in adult animals there remains significant plasticity in terms of ageing trajectories and lifespan, suggesting that targeting ageing processes directly may be a promising alternative strategy. However, to date effects of even the most efficacious pharmacological interventions are smaller than those of ageing mutations, even when targeting the same ageing pathways. Interestingly, it has been shown that simultaneously targeting multiple ageing pathways can result in lifespan benefits that are synergistic (more than additive). We have recently shown that dramatic lifespan and healthspan extension can also be archived by leveraging interactions between drugs targeting distinct subsets of the gene-regulatory network controlling ageing of C. elegans. These interventions were highly efficacious, even when animals were treated only as adults.

